# First-Principles Reaction Dynamics beyond Six-Atom Systems

**DOI:** 10.1021/acs.jpca.0c11531

**Published:** 2021-02-25

**Authors:** Gábor Czakó, Tibor Győri, Dóra Papp, Viktor Tajti, Domonkos A. Tasi

**Affiliations:** MTA-SZTE Lendület Computational Reaction Dynamics Research Group, Interdisciplinary Excellence Centre and Department of Physical Chemistry and Materials Science, Institute of Chemistry, University of Szeged, Rerrich Béla tér 1, Szeged H-6720, Hungary

## Abstract

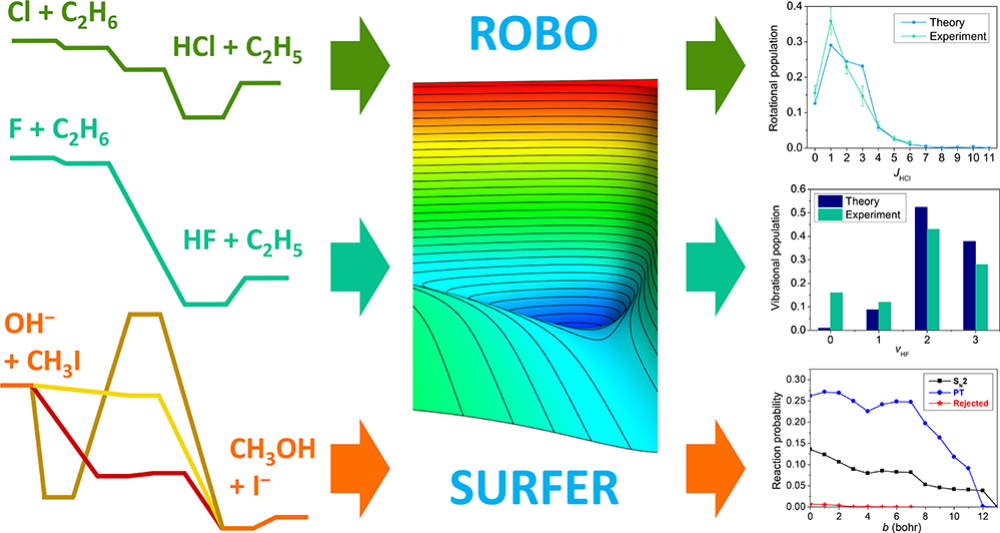

Moving beyond the six-atomic benchmark systems, we discuss the new age and future of first-principles reaction dynamics, which investigates complex, multichannel chemical reactions. We describe the methodology starting from the benchmark ab initio characterization of the stationary points, followed by full-dimensional potential energy surface (PES) developments and reaction dynamics computations. We highlight our composite ab initio approach providing benchmark stationary-point properties with subchemical accuracy, the Robosurfer program system enabling automatic PES development, and applications for the Cl + C_2_H_6_, F + C_2_H_6_, and OH^–^ + CH_3_I post-six-atom reactions focusing on ab initio issues and their solutions as well as showing the excellent agreement between theory and experiment.

## I INTRODUCTION

Accurate first-principles reaction dynamics studies began with the three-atomic H + H_2_ system in the 1970s^1^ and arrived to the six-atom reactions in the 2000s and early 2010s.^2−9^ The first-principles theoretical methodology is based on the Born–Oppenheimer potential energy surface (PES) obtained from clamped-nuclei electronic structure theory followed by nuclear dynamics computations using either quasi-classical or quantum methods. Following the pioneering work on H + CH_4_,^3^ in 2011 Czakó and Bowman developed a high-quality full-dimensional PES for the six-atomic Cl + CH_4_ reaction,^6^ which, for the first time, provided excellent agreement with the measured^10^ HCl rotational distribution, thereby confirming the fact that the quasi-classical trajectory (QCT) method can well describe the dynamics of polyatomic reactions if an accurate PES is used. Furthermore, the new PES initiated several other theoretical studies for the Cl + CH_4_ reaction,^9,11^ complementing and reproducing the crossed-beam experiments of Liu and co-workers^12,13^ and allowing quantum dynamics and/or ring-polymer molecular dynamics (RPMD) computations by the groups of Zhang,^11,14^ Yang,^13^ Guo,^13,15^ and Suleimanov.^15^ Besides H/Cl + CH_4_, the ab initio PES-based first-principles approach has been successfully applied to other similar systems, such as the F, O, Br + CH_4_ reactions.^9,14,16^ Moreover, for H + CH_4_, full-dimensional quantum dynamics computations were also performed in 2013 using the multiconfiguration time-dependent Hartree approach,^7^ thereby arriving at a similarly accurate description of six-atom systems as it was possible for three-atom reactions in the 1970s. Following the success of atom + methane simulations, in 2013 we developed the first high-level full-dimensional ab initio PES for a bimolecular nucleophilic substitution (S_N_2) reaction, namely, F^–^ + CH_3_Cl.^8^ This study opened the door for accurate reaction dynamics simulations for six-atomic ion–molecule reactions such as F^–^ + CH_3_Y [Y = F, Cl, Br, I]^17,18^ revealing a new double-inversion mechanism^19^ and front-side complex formation^20,21^ in S_N_2 reactions as well as allowing quantitative comparison with the crossed-beam experiments of the Wester group.^20,22^

The next challenge for reaction dynamics computations could be moving beyond six-atom systems. Due to the large number of degrees of freedom, in the past mostly approximative methods were applied to reactions involving more than six atoms. The two major classes of these methods are reduced-dimensional PES-based approaches and direct dynamics simulations. Clary and co-workers^23−25^ used two-dimensional quantum methods to study the dynamics of large systems, such as reactions of H atom with CH_3_NH_2_, C_2_H_6_, C_3_H_8_, C_4_H_10_, cyc-C_3_H_6_, and Si(CH_3_)_4_.^16^ Direct dynamics, which computes the potential energies and gradients on-the-fly along quasi-classical trajectories, can be successfully applied to many complex systems, as demonstrated in the case of the OH^–^ + CH_3_F/CH_3_I, F^–^(H_2_O) + CH_3_I, and F^–^ + CH_3_CH_2_I reactions by Hase and co-workers.^26−29^ However, the approximations used in the above methods may compromise their accuracy. Reduced-dimensional methods may not capture the non-intrinsic-reaction-coordinate (non-IRC) dynamics, while direct dynamics can only afford using low level of electronic structure theory and/or computing a small number of trajectories. Note that Troya^30^ used reaction-specific semiempirical Hamiltonians to improve the efficiency of the direct dynamics simulations.

In the present Perspective we discuss how to extend the accurate first-principles full-dimensional methodologies applied successfully for six-atom systems toward larger 7–10-atom reactions. This post-six-atom age of accurate full-dimensional PES-based reaction dynamics has just recently started with the investigations of the O + C_2_H_4_,^31^ OH + CH_4_,^32^ H/F/Cl/OH + CH_3_OH,^33−36^ F/Cl/O/OH + C_2_H_6_,^37−40^ OH^–^ + CH_3_I,^41^ and F^–^(H_2_O) + CH_3_I^42^ reactions. Furthermore, using empirical valence bond PESs the dynamics of the Cl + C_3_H_6_/C_5_H_12_ reactions was also investigated.^43,44^ Besides the bimolecular reactions, we should also note the pioneering work of Bowman and co-workers on CH_3_CHO photodissociation^45^ and their recent advances on efficient PES developments for many-atom systems such as CH_3_NHCOCH_3_ (*N*-methylacetamide) and NH_2_CH_2_COOH (glycine).^46^ These first-principles studies have three key steps. First, the stationary points of the PES should be characterized, thereby guiding the dynamical studies and enabling easy validation of the accuracy of key regions during the development of the fitted surface. Second, an analytical PES is developed by fitting high-level ab initio energy points. Third, the dynamics is investigated using either the QCT or time-dependent reduced-dimensional quantum dynamics methods. In our group we work on all three steps of the reaction dynamics studies and in sections II and III we give some details about the techniques used emphasizing our efficient composite approaches toward computing accurate potential energies and our software development efforts toward reducing the amount of human labor required for constructing the fitting sets for larger, high-complexity systems. Then in section III we focus on three reactions involving 7 and 9 atoms for which we developed full-dimensional ab initio PESs in 2020.^37,38,41^ The three systems represent different challenges during the PES developments from the electronic structure point of view. Cl + C_2_H_6_ is a less complicated case,^38^ for F + C_2_H_6_ the Hartree–Fock method fails in the entrance channel,^37^ and OH^–^ + CH_3_I suffers from a serious breakdown of the gold-standard CCSD(T) method.^41^ We show how to solve these problems and provide comparisons with experiments^47−49^ demonstrating the power and accuracy of first-principles reaction dynamics for post-six-atom systems. The Perspective ends with conclusions and our points of view on the future of the field in section IV.

## II METHODS

### II.A Benchmark ab Initio Characterization of the Stationary Points

Following the concept of the focal-point analysis^50^ and other thermochemical procedures such as CBS-*n*,^51^ G*n*,^52^ W*n*,^53^ HEAT,^54^ etc., we have been using a composite ab initio approach^55^ to determine the best technically feasible structures and relative energies for the stationary points of reactive PESs. Following an initial stationary-point search at the relatively cheap MP2/aug-cc-pVDZ level of theory, we optimize the minima and transition states at the explicitly correlated CCSD(T)-F12b/aug-cc-pVTZ level of theory,^56,57^ providing benchmark structures and harmonic vibrational frequencies. The best relative energies are obtained at the benchmark geometries using the following composite expression^55^
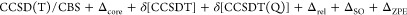
1The complete-basis-set limit of the coupled-cluster, singles, doubles, and perturbative triples method (CCSD(T)/CBS) can be approached within about 0.1 kcal/mol by explicitly correlated CCSD(T)-F12b/aug-cc-pV*n*Z computations using *n* = 4(Q) or 5 depending on system size. Core electron correlation corrections (Δ_core_) are usually obtained as difference between all-electron and frozen-core energies at the CCSD(T)-F12b/cc-pCVTZ-F12 level. Post-(T) correlation energy increments are defined as δ[CCSDT] = CCSDT – CCSD(T) and δ[CCSDT(Q)] = CCSDT(Q) – CCSDT. The CCSDT(Q) computations can be carried out with the Mrcc program package^58,59^ using the cc-pVDZ and/or aug-cc-pVDZ basis set(s) owing to the extremely high computational cost of the CCSDT(Q) method. Scalar relativistic corrections (Δ_rel_) can be obtained by difference between Douglas–Kroll^60^ and nonrelativistic all-electron energies obtained at the CCSD(T)/triple-zeta level. Spin–orbit corrections (Δ_SO_), relevant for some open-shell systems, such as reactions of halogen atoms, can be obtained with the Breit–Pauli Hamiltonian in the interacting-states approach^61^ at the MRCI+Q/aug-cc-pV*n*Z [*n* = 2(D) or 3(T)] level of theory. Zero-point energy corrections (Δ_ZPE_) needed to obtain experimentally relevant adiabatic energies from the classical ones, are usually obtained at the CCSD(T)-F12b/aug-cc-pVTZ level of theory using the harmonic oscillator approximation. More details on the above-described composite approach including references for the different ab initio methods can be found in ref (55). Note that the knowledge of the stationary points is not an essential prerequisite for PES developments; however, the stationary-point information could be helpful to show the chemically important energy range and product channels while providing benchmark data to test the accuracy of the analytical PESs.

### II.B Automatic Potential Energy Surface Development

**Figure 1:**
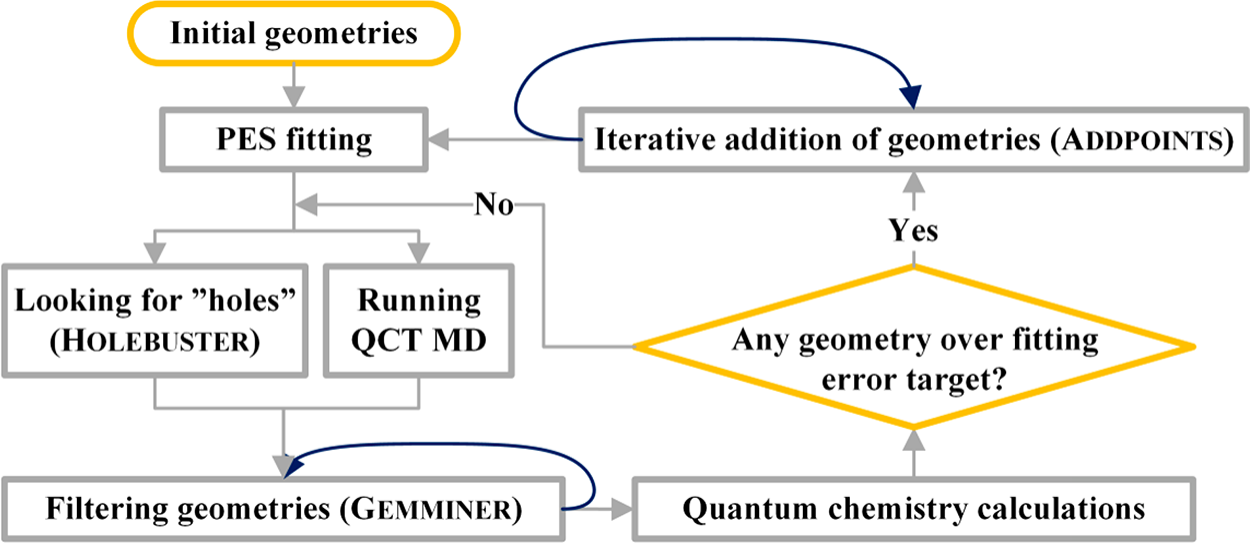
Simplified operational flowchart of the Robosurfer program system. Reprinted with permission from ref (18). Copyright 2020 American Chemical Society.

The three main steps/challenges of the analytical PES developments are the (1) selection of the nuclear configurations, (2) electronic structure computations, and (3) fitting the energy points. (1) is based on randomly displaced stationary-point geometries and/or configurations along trajectories obtained on a preliminary PES and/or by direct dynamics. (2) is performed by standard program packages like Molpro(62) using carefully chosen electronic structure methods and basis sets, such as CCSD(T)-F12b/aug-cc-pVTZ,^56,57^ which is the current state-of-the-art in the field. In the case of some systems, the use of the CCSD(T) method becomes problematic due to either Hartree–Fock convergence issues or the breakdown of the perturbative (T) correction. Examples and solutions for these problems are described in section III. Following the groundbreaking work of Braams and Bowman,^63^ (3) can be done by using the permutationally invariant polynomial (PIP) method implemented via primary and secondary invariants^63^ or an alternative implementation of PIP called the monomial symmetrization approach (MSA).^64^ The former is more efficient but currently implemented for a limited number of system types beyond six atoms, whereas the latter uses automatic code generation to be able to handle arbitrary systems; thus, we used the MSA program^64^ to fit PESs for the F/Cl + C_2_H_6_ reactions.^37,38^ While we used PIP exclusively for our PESs to date and thus have no direct experience with other fitting methods, it is also important to note that in the recent past promising neural-network-based fitting strategies started to become widespread.^33,65,66^ The above three steps are usually carried out multiple times, thereby iteratively improving the PES. Recently, we developed a program system, called Robosurfer,^18^ which automatically performs the iterative procedure as shown in Figure 1. Robosurfer first fits the initial geometries which may be generated by randomly displacing stationary-point structures and randomly scattering fragments in the reactant and product channels. Then the following steps are carried out: (a) Running trajectories and/or looking for holes (unphysical minima) by the Monte Carlo- and Newton-type minimum search method-based Holebuster subprogram. (b) Filtering geometries obtained in (a) based on a permutationally invariant exponentially weighted root-mean-square-deviation (PI-EW-RMSD) distance metric as a measure of structural similarity with the configurations in the fitting set. The larger the PI-EW-RMSD value, the more likely that the fitting error is large and the corresponding structure improves the PES. (c) Performing electronic structure computations at the selected geometries. (d) Iterative addition of the geometries to the fitting set and refitting until the largest fitting error of the spare geometries becomes less than the target accuracy or until every point is added. Robosurfer automatically goes through the above steps from (a) to (d) iteratively until the desired accuracy of the PES is achieved. The quality of the PES is checked by examining root-mean-square fitting errors (low, <1 kcal/mol), comparing stationary-point properties and one-dimensional potential cuts with benchmark data (good agreement), and most importantly, running trajectories and searching for unphysical products (zero or negligible probability), where the desired outcomes are given in parentheses.

### II.C Reaction Dynamics Computations

The analytical PESs allow efficient dynamics simulations using the QCT and/or quantum dynamics methods. The former can be done in full dimensions and the analytical PESs ensure both efficiency and accuracy via fast numerical or analytical gradient evaluations using the PESs and the high-level of ab initio theory used for the PES developments, respectively. Currently, the latter method can be used in reduced dimensions beyond six-atom systems.^16^ Quantum dynamics has the advantage of correctly describing quantum phenomena like zero-point vibration, tunneling, and resonances, but the reduced-dimensional model may compromise the proper description of complex, non-IRC reaction pathways involving the coupling of high number of degrees of freedom. Note that QCT also incorporates some quantum effects into the initial conditions and one may use the one-dimensional Gaussian binning (1GB) method^67,68^ to analyze the polyatomic products in the “quantum spirit”. Between QCT and quantum dynamics RPMD seems to be a promising tool to provide accurate results especially for rate coefficients.^15^

## III APPLICATIONS

We demonstrate the success of the first-principles methodology described in section II for post-six-atom systems by briefly discussing recent applications and results from our group on the Cl + C_2_H_6_, F + C_2_H_6_, and OH^–^ + CH_3_I reactions.^37,38,41^ As highlighted below, the three systems pose different challenges, whose solutions may be found useful in similar future investigations. In all cases we used Robosurfer(18) to automatically develop the full-dimensional PESs and the dynamics was studied by the QCT method.

### III.A CCSD(T)-F12 Success: The Cl + C_2_H_6_ Reaction

**Figure 2:**
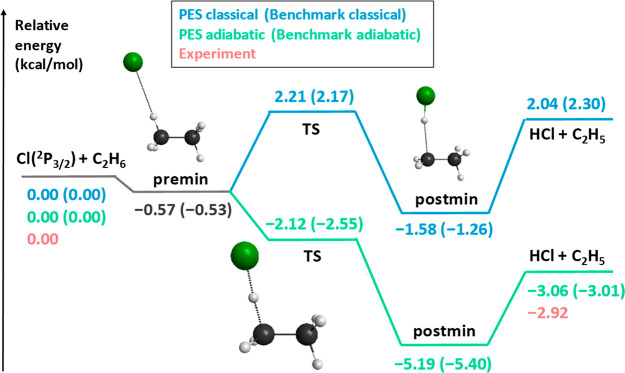
Schematic classical (blue lines without ZPE) and adiabatic (green lines with ZPE) potential energy surface of the Cl(^2^P_3/2_) + C_2_H_6_ → HCl + C_2_H_5_ reaction showing the relative energies of the stationary points corresponding to the analytical PES (ref (38)) compared with benchmark relativistic all-electron CCSDT(Q)/complete-basis-set-quality reference data (taken from ref (69) and shown in parentheses) and experiment (ATcT). Adapted with permission from ref (38). Copyright 2020 American Chemical Society.

**Figure 3:**
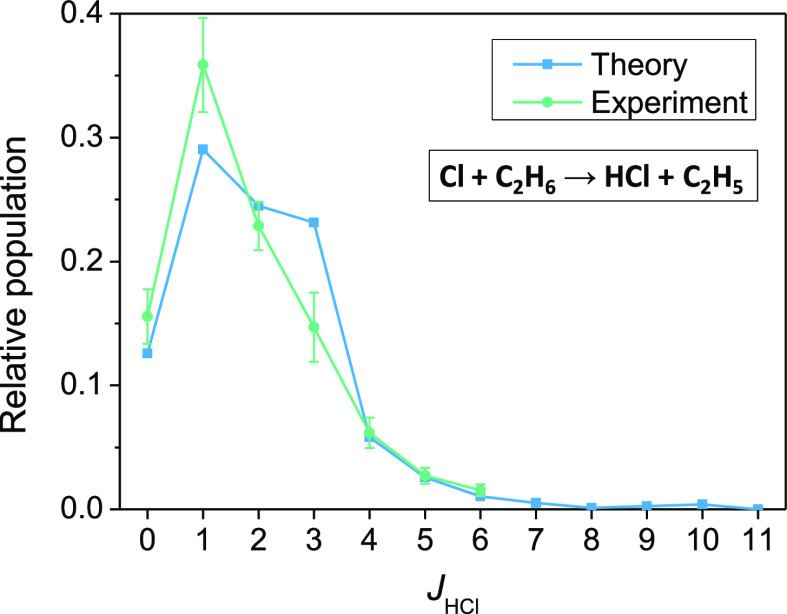
Rotational distribution for the HCl(*v* = 0) product of the Cl(^2^P_3/2_) + C_2_H_6_ reaction at 5.5 kcal/mol collision energy obtained by QCT computations on the PES of ref (38) and compared with experiment (refs (47) and (48)). Adapted with permission from ref (38). Copyright 2020 American Chemical Society.

The full-dimensional PES for the Cl(^2^P_3/2_) + C_2_H_6_ reaction was obtained by fitting 11 701 energy points with a fifth-order polynomial of Morse-like variables resulting in 3234 coefficients.^38^ The ab initio energies were computed at the UCCSD(T)-F12b/aug-cc-pVDZ + RMP2-F12/aug-cc-pVTZ – RMP2/aug-cc-pVDZ + Δ_SO_(MRCI+Q/aug-cc-pVDZ) composite level of theory, thereby obtaining spin–orbit-corrected UCCSD(T)-F12b/aug-cc-pVTZ-quality results at a significantly lower computational cost. As Figure 2 shows, the Cl(^2^P_3/2_) + C_2_H_6_ → HCl + C_2_H_5_ reaction has a small classical barrier (Δ*E*_TS_ = 2.21 kcal/mol) and is endothermic (Δ*E* = 2.04 kcal/mol) without ZPE correction, whereas the vibrationally adiabatic reaction pathway is exothermic (Δ*H*_0_ = −3.06 kcal/mol) with a submerged transition state (Δ*E*_TS_ = −2.12 kcal/mol). The relative energies of the stationary points on the PES agree with the relativistic all-electron CCSDT(Q)/CBS-quality benchmark data^69^ within 0.1–0.4 kcal/mol showing the excellent performance of the fit and the above composite ab initio method. Furthermore, dynamics simulations on the PES gave HCl rotational distributions in unprecedented agreement with Cl + C_2_H_6_ experiments^47,48^ reproducing the cold distributions with a peak at *J* = 1 as shown in Figure 3. These results demonstrate that in 2020 we reached a level of accuracy for the nine-atomic Cl + C_2_H_6_ system that was possible for the six-atomic Cl + CH_4_ reaction in 2011.^6^

### III.B Hartree–Fock Failure Solved by MRCI-F12: The F + C_2_H_6_ Reaction

**Figure 4:**
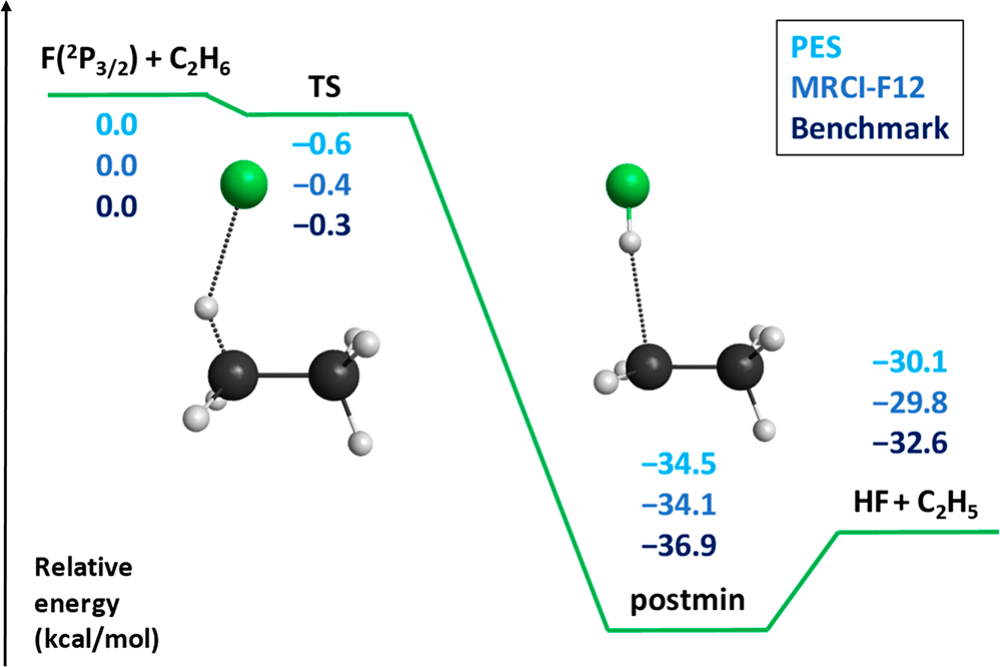
Schematic potential energy surface of the F(^2^P_3/2_) + C_2_H_6_ → HF + C_2_H_5_ reaction showing the classical (without ZPE) relative energies of the stationary points corresponding to the analytical PES (ref (37)) compared with spin–orbit-corrected MRCI-F12+Q(5,3)/aug-cc-pVDZ (ref (37)) and benchmark relativistic all-electron CCSDT(Q)/complete-basis-set-quality reference data (ref (69)). Adapted with permission from ref (37). Copyright 2020 American Institute of Physics.

**Figure 5:**
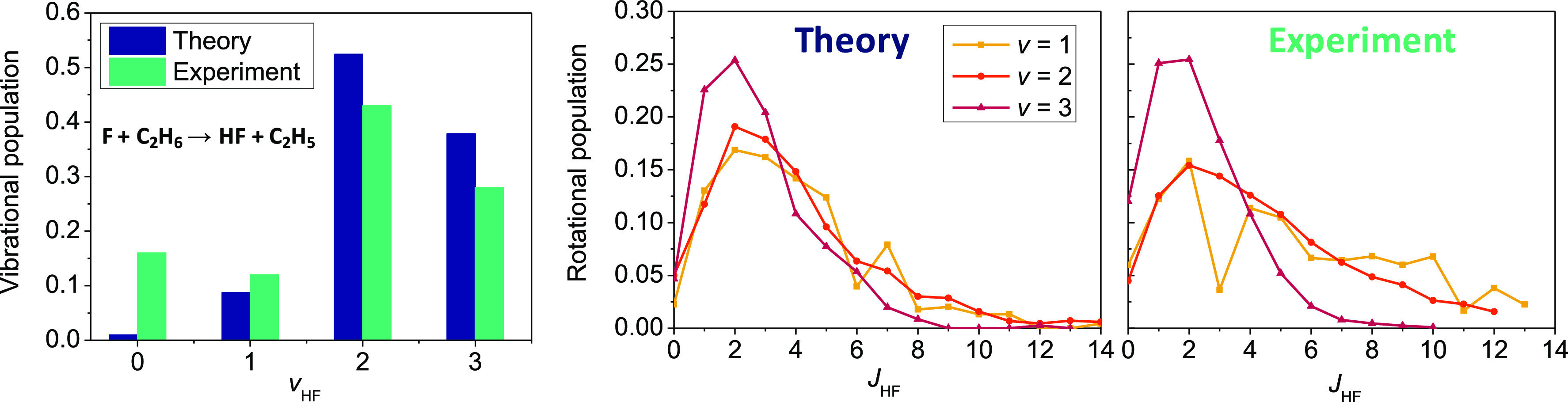
Vibrational and vibrationally resolved rotational distributions for the HF product of the F(^2^P_3/2_) + C_2_H_6_ reaction at 3.2 kcal/mol collision energy obtained by QCT computations on the PES of ref (37) and compared with experiment (ref (49)). Each distribution is normalized that the sum of the populations gives 1. The left panel is adapted with permission from ref (37). Copyright 2020 American Institute of Physics.

When we started to build the PES for the F + C_2_H_6_ reaction,^37^ we found that the Hartree–Fock method, both restricted and unrestricted, failed to converge for almost all the configurations selected by Robosurfer in the entrance channel, thereby stopping the automatic PES construction. Therefore, we chose to use the Davidson-corrected explicitly correlated multireference configuration interaction (MRCI-F12+Q) method with the aug-cc-pVDZ basis set instead to compute the ab initio energies. Based on three doublet electronic states and a minimal active space (5e,3o), we avoid the convergence problems, and using the interacting-states approach^61^ as implemented in Molpro, we obtain the energies for the ground spin–orbit state. The fifth-order PES fitting 15 178 energies reproduces the MRCI-F12+Q stationary-point data within 0.4 kcal/mol, confirming the accuracy of the fit (see Figure 4); however, the benchmark exothermicity is underestimated by 2.5 kcal/mol due to the insufficient description of dynamical electron correlation with MRCI if a small active space is used. Note that dynamical weighting in MRCI may improve the accuracy of the ground-state MRCI energy like in ref (70) in the case of the F + H_2_O reaction. The H-abstraction pathway of the F + C_2_H_6_ reaction is highly exothermic and goes through an early, slightly submerged transition state and a post-reaction complex, as seen in Figure 4. As expected for an early barrier exothermic reaction, the QCT simulations give vibrationally excited HF products with the highest populations for the *v* = 2 and *v* = 3 states, in good agreement with the experiment of Nesbitt and co-workers^49^ (Figure 5). Furthermore, the vibrationally resolved HF rotational distributions are also in excellent qualitative or even semiquantitative agreement with experiment, as shown in Figure 5, as well. Li and co-workers^35,71^ achieved similar accuracy for the rotational distributions in the case of the F/Cl + CH_3_OH reactions, showcasing again the remarkable performance of the current state-of-the-art of the field.

### III.C CCSD(T) Failure Solved by a Composite Method: The OH^–^ + CH_3_I Reaction

**Figure 6:**
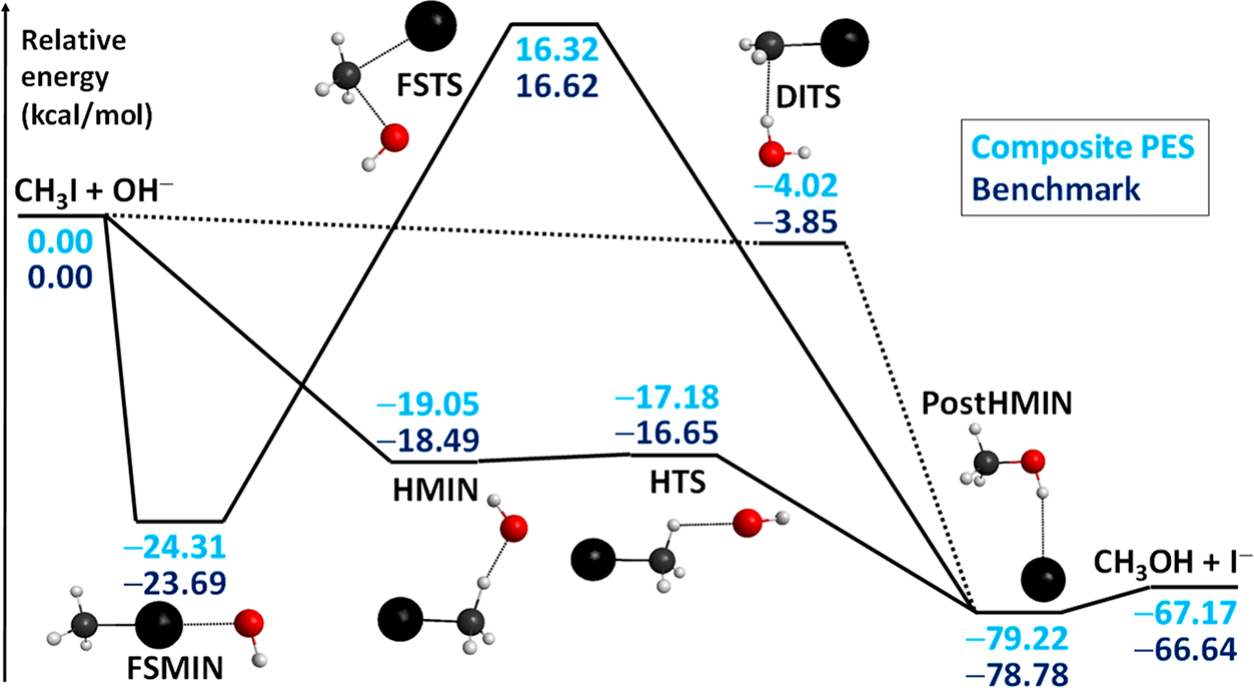
Schematic potential energy surface of the OH^–^ + CH_3_I → I^–^ + CH_3_OH S_N_2 reaction showing the classical (without ZPE) relative energies of the stationary points corresponding to the composite analytical PES (ref (41)) compared with benchmark relativistic all-electron CCSDT(Q)/complete-basis-set-quality reference data (ref (74)). The stationary-point notations are as follows: front-side minimum (FSMIN), hydrogen-bonded minimum (HMIN), hydrogen-bonded transition state (HTS), post-reaction hydrogen-bonded minimum (PostHMIN), front-side attack transition state (FSTS), and double-inversion transition state (DITS). Note that double inversion via DITS is a non-IRC pathway. Adapted with permission from ref (41). Copyright 2020 the PCCP Owner Societies.

**Figure 7:**
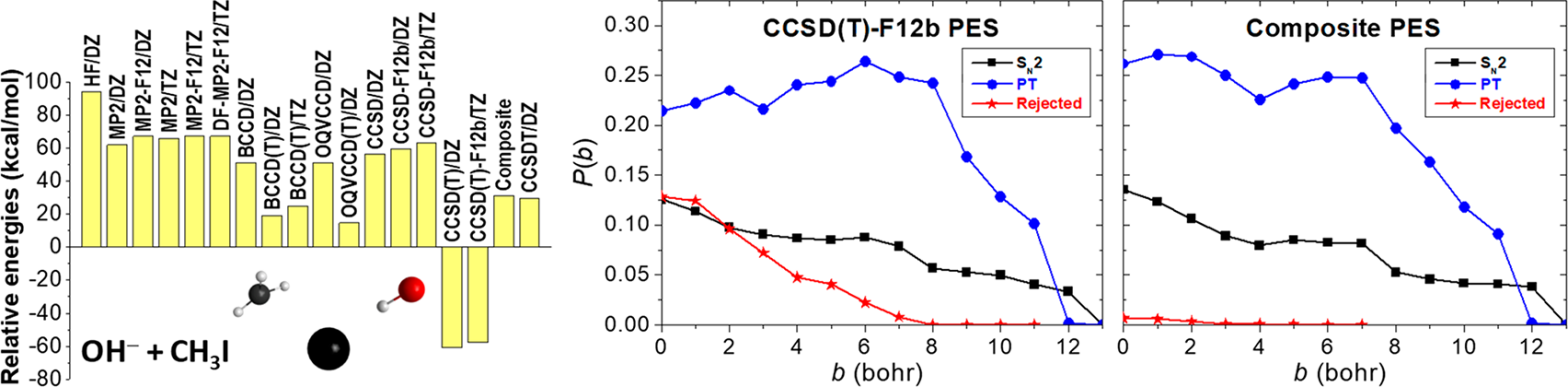
Energies of the OH^–^ + CH_3_I system relative to the reactants obtained by different ab initio methods and aug-cc-pVDZ (DZ) and aug-cc-pVTZ (TZ) basis sets corresponding to a representative nonstationary configuration taken from the fitting set where the traditional (T) approximation fails (left).^41^ The composite energy is defined as CCSD-F12b/TZ + BCCD(T)/DZ – BCCD/DZ. Reaction probabilities as a function of impact parameters for the S_N_2, proton-transfer (PT), and unphysical (rejected) channels of the OH^–^ + CH_3_I reaction obtained on a CCSD(T)-F12b/TZ PES (middle) and on a composite PES (right) at 20 kcal/mol collision energy.^41^ The left panel is adapted with permission from ref (41). Copyright 2020 the PCCP Owner Societies.

The seven-atomic OH^–^ + CH_3_I reaction has a very complex global PES, whose highly exothermic S_N_2 pathways are shown in Figure 6. The nontraditional Walden-inversion pathway goes through submerged H-bonded minima (HMIN and PostHMIN) and a transition state (HTS), whereas retention can occur via a relatively high front-side attack barrier (FSTS) or a submerged double-inversion pathway (DITS). Note that as Hase and co-workers pointed out for the Walden inversion of OH^–^ + CH_3_F^26^ and the double inversion of F^–^ + CH_3_I,^72^ the present reaction may not follow the IRC pathways. Besides the S_N_2 channel, proton transfer forming H_2_O + CH_2_I^–^ can also occur via a barrier-less exothermic pathway.^27^ In the past only direct dynamics simulations could be performed for the OH^–^ + CH_3_I reaction due to the lack of an analytical PES.^27^ Recently, we developed such a PES utilizing the Robosurfer program system.^41^ This PES development was not without complications, because the CCSD(T)-F12b PES gave many unphysical trajectories due to the breakdown of the perturbative (T) approximation. As shown in Figure 7 for a representative configuration with positive energy relative to the reactants, the CCSD(T) and CCSD(T)-F12b methods give erroneous, large negative relative energies of about −60 kcal/mol, whereas CCSD and CCSD-F12b provide results around +60 kcal/mol and the full CCSDT method, which does not use the perturbative approximation for triples, also provides a positive energy of 30 kcal/mol. We found that the Brueckner-orbitals-based BCCD(T) method^73^ gives reasonable energies for the problematic structures; therefore, we proposed a composite energy expression of CCSD-F12b/aug-cc-pVTZ + BCCD(T)/aug-cc-pVDZ – BCCD/aug-cc-pVDZ, which ensures the fast basis-set convergence with CCSD-F12b and incorporates the (T) correlation with the more robust Brueckner-type coupled-cluster approach. As seen in Figure 7, this composite method provides CCSDT-quality relative energies at significantly less computational cost and, as Figure 6 shows, the composite PES gives stationary-point relative energies in good agreement with the relativistic all-electron CCSDT(Q)/CBS-quality benchmark data^74^ with a maximum deviation of 0.53 kcal/mol. Figure 7 also shows the reaction probabilities on the CCSD(T)-F12b PES and on the composite PES. As seen, the S_N_2 and proton-transfer opacity functions are similar on the two different PESs, which is comforting; however, the CCSD(T)-F12b PES results in unphysical trajectories (energetically nonavailable products) with significant probabilities, for example, 13% at zero impact parameter, whereas the unphysical probabilities become negligible on the composite PES. Thus, it appears that the present composite method will be useful for PES developments for similar systems, especially where homolytic C–I bond cleavage may take place.

## IV CONCLUSIONS AND FUTURE DIRECTIONS

First-principles theory has arrived to a new age where accurate simulations can be performed for chemical reactions involving more than six atoms. The three major steps of the reaction-dynamics methodology are (1) the benchmark ab initio characterization of the stationary points, (2) potential energy surface developments, and (3) reaction dynamics simulations. We propose high-level composite methods for (1), the use of the Robosurfer program package^18^ for (2), and QCT or reduced-dimensional time-dependent quantum methods for (3). Since we reported Robosurfer in 2020,^18^ we have already developed automatically three PESs for post-six-atom reactions, namely, for the Cl + C_2_H_6_, F + C_2_H_6_, and OH^–^ + CH_3_I systems.^37,38,41^ Furthermore, the benchmark ab initio mapping of the complex PESs for several other systems, such as OH^–^ + CH_3_F,^74^ NH_2_^–^ + CH_3_I,^75^ F^–^ + CH_3_CH_2_Cl,^76^ and OH + C_2_H_6_^77^ with 7, 8, 9, and 10 atoms, respectively, were recently published in our group and full-dimensional PES developments are underway. We expect that in the new decade automatic, perhaps black-box, PES developments for post-six-atom reactions will become widespread allowing accurate dynamical investigations of multichannel reactions as prototypes of complex reaction networks. Full-dimensional quantum dynamics treatment may be extended for seven-atom systems, new reduced-dimensional quantum models may be developed for 7–10-atom reactions, and the RPMD technique may be utilized for bimolecular reaction dynamics beyond its usual kinetics applications. Of course, reaction dynamics studies cannot avoid electronic structure theories, where the explicitly correlated F12 methods^56^ started to become the state-of-the-art in the 2010s. We may use MRCI where single-reference methods fail as we showed for F + C_2_H_6_;^37^ however, approaching the accuracy of CCSD(T) with MRCI for describing nonstatic electron correlation is usually prohibitive or requires a very large active space. If the perturbative (T) approximation breaks down, a Brueckner-type coupled-cluster-based composite method could be useful as shown for OH^–^ + CH_3_I.^41^ Furthermore, quasi-variational coupled-cluster methods were developed recently,^78^ which also showed promising behavior in our dynamics studies.^41,79^ Finally, we emphasize that several first-principles reaction dynamics studies proved that theory is capable of providing results in excellent agreement with experiment. We hope that new experiments will also be carried out for post-six-atom reactions, thereby moving the field forward hand in hand with theory.
